# Under-nutrition and associated factors among children on ART in Southern Ethiopia: a facility-based cross-sectional study

**DOI:** 10.1186/s13052-021-01154-w

**Published:** 2021-10-11

**Authors:** Chalie Marew Tiruneh, Belete Gelaw Walle, Tigabu Desie Emiru, Nigusie Selomon Tibebu, Moges Wubneh Abate, Adane Birhanu Nigat, Amsalu Belete, Eyasu Alem, Tadele Lankrew, Kirubel Eshetu

**Affiliations:** 1 Department of Pediatrics and Child Health Nursing, College of Health Sciences, Debre Tabor University, P.O.Box 272, Debre Tabor, Ethiopia; 2 Department of Pediatrics and Child Health Nursing, College of Health Science and Medicine, Wolaita Sodo University, Wolaita Sodo, Ethiopia; 3 Department of Adult health Nursing, Debre Tabor University, Debre Tabor, Ethiopia; 4 Department of Psychiatry, School of medicine , Debre Tabor University, Debre Tabor, Ethiopia; 5 Department of Adult health Nursing, Wolaita Sodo University, Wolaita Sodo, Ethiopia

**Keywords:** Antiretroviral therapy, Children, HIV, Nutritional status

## Abstract

**Background:**

Malnutrition is very common in HIV-infected individuals. Even though data from different settings are necessary to tackle it, pieces of evidence are limited especially in the case of the nutritional status of HIV-infected children. Hence, this study aims to assess the nutritional status and associated factors among children on antiretroviral therapy.

**Methods:**

An institutional-based cross-sectional study was conducted among 383 HIV-positive children in Southern Ethiopia. Data were collected using an interviewer-administered questionnaire and anthropometry measurement. Data were coded and entered into Epi-Data Version 3.1 and analyzed using SPSS Version 25. Bi-variable and multi-variable binary logistic regression models were used to identify factors associated with nutritional status and variables with *p*-values <0.05 in multi-variable logistic regression were considered as statistically significant.

**Results:**

The prevalence of wasting among HIV-positive children in Southern Ethiopiaselected Hospitals was 36.3% (95% CI, 31.6–41.0) while stunting on the same study population was 5.5% (95% CI, 3.4–7.8). Rural residence, lack of maternal education, low CD4 counts (< 500), using an unprotected water source, having a non-biological mother and recurrent oral lesion were significantly associated with wasting. Furthermore, history of hospital admission, recurrent oral lesion, low CD4 counts (< 500), advanced WHO clinical stage were statically associated with stunting with *p*-value < 0.05.

**Conclusion:**

This study found that the prevalence of under-nutrition among HIV-positive children in Ethiopia was significantly high. Therefore, timely identification and monitoring of nutritional problems should be necessary to enhance the effectiveness of ART treatment and to prevent further related complications.

## Background

The pandemic of the human immunodeficiency virus (HIV) is one of the major public health problems and is associated with a range of long and short-term consequences [1]. At the end of 2019, approximately 38.0 million people were living with HIV globally, of which 1.8 million were children (age 0–14 years) [2]. Ethiopia is one of the Sub-Saharan Africa (SSA) countries, which suffer from the global burden of HIV- infection. By the end of 2018, an estimated 56,514 children under the age of 15 were living with HIV, of which, around 2994 were newly infected with HIV [3].

Malnutrition is one of the major causes of death for HIV-positive children [4]. Human Immune deficiency Virus (HIV) infection and malnutrition often coexist, which increases the risk of morbidity and mortality [5]. Malnourished children have lower resistance to infection and are more likely to die from a common childhood illness. Children living with the human immunodeficiency virus (CLHIV) are physically stunted and underweight compared to non-infected children [6].

Maintaining good nutritional status remains a very challenging issue for HIV-positive children. The problem is related to inadequate dietary intake, the effect of anti- retro Virus therapy (ART), and the HIV infection itself [5]. People with HIV/AIDS often do not eat enough as the illness and the drugs are taken alter the food taste, decrease appetite, and inhibit the body rate of food absorption.

In Ethiopia, there are only a few studies that are conducted to assess the nutritional status of CLHIV [5–9]. However, to the best of our knowledge, there was no study conducted to explore the nutritional status and associated factors among HIV-positive children (15 years of age) in the study area. Current and up-to-date evidence regarding nutritional status in HIV-positive children is essential for policymakers and clinicians to take appropriate actions. Therefore, the findings of this study will highlight the magnitude and associated factors of malnutrition among HIV-positive children with implications to improve health workers’ interventions, to ensure treatment effectiveness, and to provide its contribution in supplying baseline information for the reduction of HIV-related morbidity and mortality of children.

## Methods

### Study area, design, and period

An institutional-based cross-sectional study was conducted from February to March 2021 among HIV-infected children on ART in Southern Ethiopia. The study was carried out in three selected governmental hospitals (i.e., Otona Teaching and Referral Hospital, Halaba District Hospital, and Duramie General Hospital). These hospitals provide service for more than 6 million people in the Region. All three hospitals provide chronic HIV care and follow-up services for HIV-infected clients. Nowadays, there are approximately 579 children (< 15 years of age) receiving ART follow-up service in these hospitals.

### Study participants, sample size, and sampling technique

All confirmed HIV-positive children (aged < 15 years) taking ART in Southern Regional State governmental hospitals were the target population. All HIV-infected children who had ART follow up at the selected hospitals were the study population. However, children with incomplete baseline medical information were excluded. Furthermore, a child who does not have a caretaker or parents to undertake the consent, caretakers diagnosed to have mental problems, or children who have physical malformation and are seriously ill were excluded from the study.

The minimum required sample size was determined using a single population proportion formula [10]. To calculate our sample size, the following statistical assumptions were considered: 60.2% proportion (p) of malnutrition from a study done in East and West Gojam Zones, Amhara, Northwest, Ethiopia [8]; 5% margin of error; 10% non-response rate; and 95% confidence intervals (CI).

$$ \mathrm{n}=\frac{{\left(\mathrm{Za}/2\right)}^2\mathrm{p}\left(1-\mathrm{p}\right)}{{\left(\mathrm{d}\right)}^2}=\frac{(1.96)^2\ 0.6\left(1-0.6\right)}{(0.05)^2}=368.64\sim 369 $$

Where, n = the required sample size, Zα/2 = Standard normal variation for type 1 error, p = prevalence (0.5) & d = Margin of sampling error tolerated (0.05).

The calculated sample size was 369. After considering a 10% non-response rate, the final sample size of our study was 406.

This study was conducted in three randomly selected governmental hospitals. From the beginning, a sampling frame was prepared using the patient’s medical registration number from each hospital’s ART registration logbook. Then, the total sample sizes were allocated proportionally for each hospital. Finally, study participants were taken from each of the three selected hospitals using a computer-generated simple random sampling technique.

### Data collection tool and procedure

The data abstraction checklist was developed from the current Ethiopian Federal Ministry of Health ART clinic intake and follow-up forms. Data were collected through anthropometric measurement, face-to-face interviews, and clinical records reviewed by trained health professionals. Training about the objectives of the study, the contents of the tool, and data collection procedures was given for data collectors and supervisors for 1 day. The pretest was carried out at Sodo health center. During the data collection time, caregivers who had a malnourished child were linked to therapeutic feeding centers. Besides, weight and height were measured for each study participant, and nutritional advice was given to all caregivers. The assigned supervisors and principal investigator closely monitored and supervised the whole data collection process.

### Operational definitions

Under-nutrition: - was defined when the children having either W/H or H/A or W/A z-score < −2SD of the median value of WHO standard [11, 12].

### Data management and statistical analysis

The consistency and completeness of the collected data were examined during data management and analysis. Data were entered into Epi Data Version 3.1 and analysis was done using Statistical Package for Social Science (SPSS) Version 25. The anthropometric measurements were converted into Z-scores using WHO Anthro Plus software version 3.2.2. Frequencies and cross-tabulations were used to check for missed values of variables and to describe the study population concerning relevant variables. Moreover, percentages, proportions, and summary statistics (mean, median) were used to summarize the study population characteristics. Binary logistic regression analysis was implemented to assess the association of factors against the outcome variable. Variables with *p*-values < 0.25 in the bivariable analysis were entered into the final model to control the effects of confounders and identify significant factors. Adequacy of the model to fit the outcome variable with the predictors was checked using the Hosmer-Lemeshow test for goodness of fit. In the multivariable analysis, variables with p-values less than 0.05 at 95% CIs were considered statistically significant factors. Finally, the strength and the direction of association were assessed using odds ratios with their correspondence 95% CIs.

## Results

### Socio-demographic characteristics of study participants

Out of 406 study participants, 383 were included in this study with a response rate of 94.3%. Nearly half of the study participants’ 193 (50.4%) were boys and 157 (41%) were from rural areas. Children age less than 60 months were 124 (32.4%), while 52 (13.6%) of the study participants were between 60 and 120 months. The majority 282 (73.6%) of caretakers were unmarried, and most 209 (54.6%) of the caretakers were unable to read and write. Among the caretakers, 152 (39.7%) were daily laborers, and more than half 146 (61.9%) of them have greater than four families in the house they live in (Table 1).

**Table 1:** Socio demographic characteristics of HIV positive children under 15 years of age attending ART care at public health institutions in Southern Ethiopia, 2021

Variable	Category	Frequency	Percent
Age	< 60 months	124	32.4
	60 to 120 month	52	13.6
	121 to 180 month	207	54.0
Sex	Male	193	50.4
	Female	190	49.6
Religion	Protestant	107	27.9
	Orthodox	76	19.8
	Muslim	76	19.8
	Catholic	58	15.1
	Other	66	17.2
Ethnicity	Kenbata	64	16.7
	Wolayta	258	67.4
	Tigre	14	3.7
	Oromo	28	7.3
	Amhara	19	5.0
Residence	Urban	116	30.3
	Rural	157	41.0
	Refugee	110	28.7
Marital status of care takers	Unmarried	282	73.6
	Married	101	26.4
Care taker relation with the child	Biological mother	92	24.0
	other	291	76.0
Educational status of Care giver	unable to read and write	209	54.6
	able to read and write	174	45.4
Occupational status mother	Government employed	45	11.7
	NGO employed	41	10.7
	Merchant	126	32.9
	Daily Laborer	152	39.7
	Other	19	5.0
Monthly family income (Ethiopian Birr)	< 1500	77	20.1
	1500 to 3000	126	32.9
	> 3000	180	47.0
Family size	< 4	146	38.1
	> = 4	237	61.9

### Environmental related characteristics

In this study, about 52.7% of study participants used unprotected drinking water sources. The majority (40.5%) of the respondents used an open field waste disposal system and 54.8% of them did have not any nutritional support (Table 2).

**Table 2:** Environmental related factors of the study population for HIV positive children under 15 years of age attending ART care at public health institutions in Southern Ethiopia, 2021

Variable	Category	Frequency	Percent
Source of water	protected	181	47.3
	unprotected	202	52.7
Waste disposal system	Open field	155	40.5
	Burned	130	33.9
	Other (specify)	98	25.6
Availability of nutritional support	Yes	173	45.2
	No	210	54.8
Toilet utilization	Not using toilet	50	13.1
	Use toilet facility	333	86.9

### Clinical related characteristics

One hundred twenty-nine (33.7%) of study participants had less than normal birth weight. Almost one-third of participants (32.9%) were categorized to WHO clinical stage III and 35.5% of them had CD4 count less than 350 cells/mm3. Slightly more than one-third, 138 (36%) of children were experienced opportunistic infection and the commonest, 248 (64.8%) was oral lesion. Half, 192 (50.1%) of study participants had a history of admission. Of all respondents, 122 (31.9%) of study participants had poor adherence levels during their last follow-up time (Table 3).

**Table 3:** clinical related factors of the study population for the study of nutritional status and associated factors among HIV positive children under 15 years of age attending ART care at public health institutions in Southern Ethiopia, 2021

Variable	Category	Frequency	Percent
Birth weight	< 2.5 kg (Less than Normal)	129	33.7
	2.5 kg and above (Normal)	142	37.1
	I don’t know	112	29.2
child had diarrhea	Yes	166	43.3
	No	217	56.7
Diagnosis of any disease during pregnancy of this child	Yes	177	46.2
	No	206	53.8
Recurrent oral lesion	Yes	248	64.8
	No	135	35.2
History of admission	Yes	194	50.7
	No	189	49.3
WHO clinical stage	Stage 1	121	31.6
	stage 2	38	9.9
	stage 3	98	25.6
	stage 4	126	32.9
ARV drug toxicity	Yes	14	3.7
	No	369	96.3
Co -morbid illness	Yes	138	36.0
	No	245	64.0
Adherence to the drug	poor	122	31.9
	Faire	120	31.3
	Good	141	36.8
Current CD4 count	> = 500	162	42.3
	350–499	85	22.2
	200–349	136	35.5

### Prevalence of under-nutrition

**Fig. 1:**
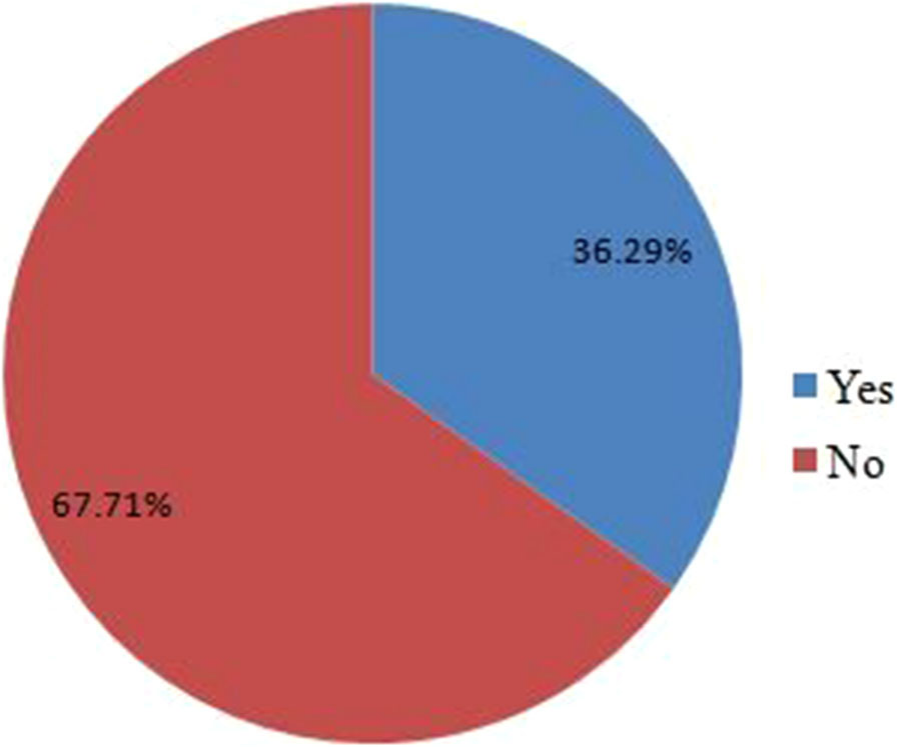
Prevalence of wasting among HIV positive children under 15 years of age attending ART at public health institutions in Southern Ethiopia

**Fig. 2:**
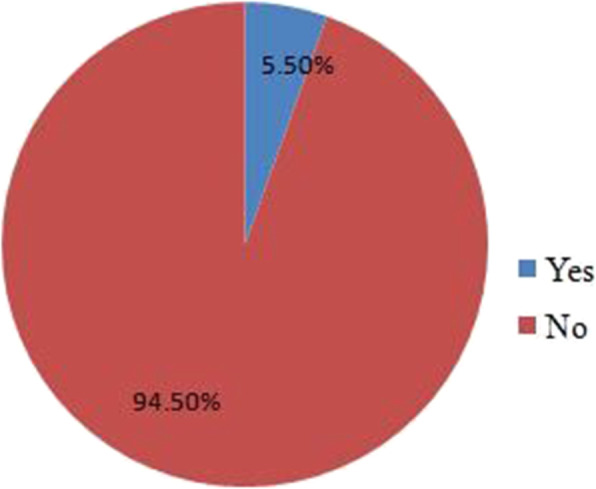
Prevalence of stunting among HIV positive children under 15 years of age attending ART care at public health institutions in Southern Ethiopia, 2021

The overall prevalence of wasting was 36.3%(95% CI: 31.6, 41.0) (Fig. 1). Moreover, the prevalence of stunting in the same study population was 5.5%(95% CI: 3.4, 7.8) (Fig. 2)*.*

### Factors associated with wasting

This study examined the relationship between the different participant characteristics and the presence of wasting. In the bivariate analysis residency, age, maternal education, WHO clinical stage, source of water, marital status of caretakers, having diarrhea in the last 2 weeks, current CD4 count, having oral thrush, and caretakers relation with the child were found to be eligible variable for adjustment in multivariable analysis. After adjusting for possible confounders in the multivariable analysis residency, maternal education, current CD4 count, having recurrent oral lesion, marital status of the mother, caretaker’s relation with child, and a source of water were remained to show statistically significant association with wasting of HIV positive children.

The likelihood for the presence of wasting was about four times more among the HIV-infected children who live in the rural area (AOR 4.083; 95% CI = 1.985–8.400) compared to those who live in urban. Regarding maternal education, wasting was about nine times (AOR = 9.329; 95% CI = 5.017–17.348) more likely to occur in those HIV-infected children who have the mother unable to read and write as compared to those who were able to read and write. HIV-infected children who had a current CD4 count of less than 500 were five times more likely to have wasting (AOR = 4.911; 95% CI = 2.325–10.369) as compared to those HIV positive children who had CD4 count greater than 500 cell/mm^3^. The likelihood for the presence of wasting was about three times more among the HIV infected children who use unprotected water source (AOR = 3.216; 95% CI = 1.787–5.788) compared to who uses protected water source. Moreover, HIV positive children those who have non-biological mother were more likely (AOR = 4.172; 95% CI = 1.894–9.190) to have wasting than those whose caretakers were biological mothers. Regarding recurrent oral lesions, HIV-positive children who have recurrent oral lesions were about two times more likely (AOR = 2.221; 95% CI = 1.169–4.219) to have wasting than those who did not complain of the oral lesion (Table 4).

**Table 4:** bivariable and multivariable analysis of the study population HIV positive children under 15 years of age attending ART care at public health institutions in Southern Ethiopia, 2021

Variable and Category	Wasting		Bi-variable logistic regression analysis		Multi-variable logistic regression analysis	
	Yes, N (%)	No N (%)	*p*-value	COR with 95%CI	AOR with 95% CI	*P*-Value
Residency						
Rural	79 (56.8%)	80 (32%)	0.02	2.25 (1.362–3.720)	**4.083 (1.985–8.400)**	0.000*
Refuge	24 (17.3%)	86 (35.2%)	0.11	0.62 (0.341–1.129)	1.265 (0.582–2.749)	0.553
Urban	36 (25.9%)	78 (32.8%)		1		
Age in month						
< 60	51 (36.7%)	73 (29.9%)	0.129	1.428 (0.901–2.263)	0.944 (0.478–1.865)	0.868
60–120	20 (14.4%)	32 (13.1%)	0.446	1.278 (0.681–2.398)	0.553 (0.232–1.319)	0.182
121–180	68 (48.9%)	139 (57.0%)		1		
Mother education						
unable to read and write	104 (74.8%)	70 (28.7%)	0.000	7.386 (4.603–11.852)	**9.329 (5.017–17.348)**	0.000*
Able to read and write	35 (25.2%)	174 (71.3%)		1		
WHO Clinical stage						
Stage IV	50 (36.0%)	67 (27.5%)	0.002	2.359 (1.368–4.070)	1.370 (0.672–2.795)	0 .386
Stage III	43 (30.9%)	61 (25.0%)	0.005	2.228 (1.271–3.908)	0.703 (0.320–1.544)	0.380
Stage II	15 (10.8%)	18 (7.4%)	0.017	2.634 (1.189–5.836)	1.075 (0.331–3.489)	0.905
Stage I	31 (22.3%)	98 (40.2%)	1	1		
Having diarrhea						
Yes	98 (70.5%)	119 (48.8%)	0.000	2.511 (1.613–3.909)	1.779 (0.977–3.239)	0.060
No	41 (29.5%)	125 (51.2%)		1		
Marital status						
Unmarried	59 (42.4%)	42 (17.2%)	0.000	3.547 (2.211–5.692)	1.855 (0.970–3.549)	0.062
Married	80 (57.6%)	202 (82.8%)		1		
Current CD4 count						
> 500	36 (25.9%)	126 (51.6%)		1		
350–499	48 (34.5%)	37 (15.2%)	0.000	4.541 (2.577–8.002)	**4.911 (2.325–10.369)**	0.000*
200–359	55 (39.6%)	81 (33.2%)	0.001	2.377 (1.435–3.936)	1.771 (0.909–3.451)	0.093
Oral lesion						
Yes	109 (78.4%)	139 (57.0%)	0.000	2.745 (1.703–4.422)	**2.221 (1.169–4.219)**	0.015*
No	30 (21.6%)	105 (43.0%)		1		
Source of water						
Unprotected	104 (74.8%)	98 (40.2%)	.000	4.427 (2.793–7.017)	**3.216 (1.787–5.788)**	0.000*
Protected	35 (25.2%)	146 (59.8%)		1		
Care takers relation						
Biological mother	13 (9.4%)	79 (32.4%)	1			
Other than mother	126 (90.6%)	165 (67.6%)	0.000	4.641 (2.470–8.720)	**4.172 (1.894–9.190)**	0.000*

1 = reference

*p-value less than 0.05

### Factor associated with stunting

This study also examined the relationship between the different participant characteristics and the presence of stunting. In the bivariable analysis sex, history of hospital admission, maternal educational status, marital status of caretaker, current CD4 count, having recurrent oral lesion and current CD4 count of the child were found to be eligible variables for adjusting in multivariable analysis. After adjusting for possible confounders in the multivariable analysis history of hospital admission, having recurrent oral lesion, WHO clinical stage, and current CD4 count were statistically significant association with stunting of HIV positive children.

The likelihood for the presence of stunting was about five times more among the HIV-infected children who had a history of hospital admission (AOR 4.938; 95% CI = 1.629–14.966) compared to those who did not have a history of hospital admission. HIV-infected children who had a current CD4 count of less than 500 were more than three times more likely to have stunting (AOR = 3.490; 95% CI = 1.016–11.985) as compared to those HIV positive children who had CD4 count greater than 500 cell/mm^3^. Regarding recurrent oral lesions, HIV-positive children who have recurrent oral lesions were about four times more likely (AOR = 3.932; 95% CI = 1.094–14.126) to have stunting than those who did not complain of the oral lesion. Moreover, the likelihood of stunting was about four times more (AOR = 3.982; 95% CI = 1.113–14.251) in WHO clinical stage three as compared to WHO clinical stage four (Table 5).

**Table 5:** bivariate and multivariate analysis of the study population for HIV positive children under 15 years of age attending ART care at public health institutions in Southern Ethiopia, 2021

Variable and Category	Stunting		Bi-variable logistic regression analysis		Multi-variable logistic regression analysis	
	Yes, N (%)	No N (%)	*p*-value	COR with 95%CI	AOR with 95% CI	*P*-Value
Sex of the child						
Male	149 (66.7%)	179 (49.4%)		1		
Female	7 (33.3%)	183 (50.6%)	0.132	2.045 (0.806–5.184)	1.489 (0.535–4.145)	0.446
History of admission						
Yes	16 (76.2%)	178 (49.2%)	0.022	3.308 (1.187–9.220)	**4.938 (1.629–14.966)**	0.005*
No	5 (23.8%)	184 (50.8%)		1		
WHO Clinical						
Stage IV	4 (19.0%)	122 (33.7%)	.954	0.959 (0.234–3.924)	0.928 (0.217–3.956)	0.919
Stage III	3 (14.3%)	88 (24.3%)	.048	3.324 (1.009–10.948)	**3.982 (1.113–14.251)**	0.034*
Stage II	10 (47.6%)	35 (9.7%)	.243	2.507 (0.535–11.740)	3.168 (0.630–15.946)	0.162
Stage I	4 (19.0%)	117 (32.3%)	1			
Can read and write						
Yes	8 (38.1%)	201 (55.5%)	0.125	2.029 (0.821–5.014)	1.443 (0.536–3.885)	0.468
No	13 (61.9%)	161 (44.5%)		1		
Marital status						
Unmarried	8 (38.1%)	93 (25.7%)	0.215	1.780 (0.715–4.430)	1.239 (0.442–3.472)	0.683
Married	13 (61.9%)	269 (74.3%)		1		
Current CD4 count						
> =500	5 (23.8%)	157 (43.4%)		1		
350–499	8 (38.1%)	77 (21.3%)	0.044	3.262 (1.033–10.305)	**3.490 (1.016–11.985)**	0.047*
200–359	8 (38.1%)	128 (35.4%)	0.247	1.962 (0.627–6.145)	2.589 (0.774–8.663)	0.123
Having recurrent oral lesion						
No	3 (14.3%)	143 (39.5%)		1		
Yes	18 (85.7%)	219 (60.5%)	.031	3.918 (1.133–13.542)	**3.932 (1.094–14.126)**	0.036*

1 = reference

*p-value less than 0.05

## Discussion

Malnutrition is very common in HIV-infected individuals by affecting food intake, altering digestion and absorption, altering metabolism, and increasing energy needs. In the current study, the magnitude of Wasting among the pediatric age group living with HIV/AIDS was 36.3%(95% CI = 31.6–41). This is in line with the study in Nigeria in which the proportion of wasting was 33.5% [13].

But, our finding is higher when compared to the studies conducted in Eastern Ethiopia (28.2%) [5], Oromia (21.8%) [7], Cameron (18.4%) [14], Nigeria (Central and West-African HIV-care (9%) [15] and Tanzania (9.4%) [16]. The present study also revealed that the magnitude of stunting among the pediatric age group living with HIV/AIDS was 5.5%(95% CI = 3.4–7.8). This is lower than the studies conducted in Oromia (13.4%) [7], Eastern Ethiopia (24.7%) [5], and Cameron (63.6%) [14]. The discrepancy between these studies could be due to the difference in study approach (population and hospital-based), study population (age group), and sampling technique.

A study among Non-HIV-positive children in southern Ethiopia revealed the magnitude of wasting was 28.2%, 25.2 and 9% [17–20]. This result is lower than the finding of the current study. The reason for the discrepancy of wasting among HIV positive and HIV negative children is HIV positive children are more susceptible to undernutrition by decreasing intake, altering digestion, absorption, and metabolism as well as by increasing energy need secondary to infection [21].

In this study, children who had recurrent oral lesions were more likely to be wasting. This is in line with previous studies conducted in Ethiopia-Gojam, Cameron, and North Wollo [8, 14, 22]. This is due to children with oral lesions have difficulty of swallowing, which reduces the amount of food intake that leads to nutritional imbalance less than body requirement.

This study revealed that children in the advanced WHO clinical stages were more likely to be wasted. This is in line with previous studies conducted in Eastern Ethiopia children [5, 22]. This can be explained by the fact that HIV-positive people who have advanced stage of the disease are more vulnerable to opportunistic infections, making them susceptible to undernutrition by decreasing intake, by altering digestion, absorption, and metabolism as well as by increasing energy need [21].

In the present study, children who lived in rural areas were four times more likely to be wasted. This is in line with a study conducted in eastern Ethiopia [5]. This is due to low access to health facilities for the early management of malnutrition, decreased level of awareness of balanced diet, and lack of infrastructure to access the health facilities.

This study also revealed that wasting is associated with the educational status of the mother. This can be explained by, mothers who are unable to read and write are more prone to have knowledge deficits secondary to being unable to read literature and magazines that deal with the nutritional requirement of children infected by HIV/AIDS. These mothers may also have a lack of awareness on the early management of malnutrition.

Finding from this study also revealed that the prevalence of wasting was more likely in those HIV-positive children who use unprotected water sources. The possible explanation for this is those HIV-positive children who use unprotected water sources are more vulnerable to develop a water-borne disease like worms because of their immune-compromised status, which predisposes them to have malnutrition [23, 24].

Finding from this study also revealed that the prevalence of wasting was more likely in those HIV-positive children who had no biological caretakers. This is due to children whose caretakers are non-biological mothers are less likely to get breastfeeding and they are also more prone to have poor drug adherence and good nutritional supplementation.

In this study history of hospital admission was associated with stunting. This is due to the reason that a history of hospital admission among HIV-positive children is an indicator of opportunistic infection secondary to low immune status, which decreases food intake.

This study revealed that those HIV-positive children who have WHO stage III are more likely to have stunting as compared to those with WHO clinical stage one. This is supported by the study conducted in Eastern Ethiopia [5]. This can be explained by the fact that HIV-positive people who have advanced stage of the disease are more vulnerable to opportunistic infections, making them susceptible to undernutrition by decreasing intake, by altering digestion, absorption, and metabolism as well as by increasing energy need [25].

According to the finding of this study, having a CD4 count between 350-499cell/mm3 is more likely to develop stunting as compared to those whose CD4 count is greater than 500cell/mm3. This is explained by those whose CD4 count is between 350-499cell/mm3 may have low attention of care by health workers compared to those who have CD4 count less than 350cell/mm3 [25].

In this study, children who had recurrent oral lesions were more likely to be stunting. This is because children with oral lesions have difficulty swallowing which leads to nutritional imbalance less than body requirement.

### Limitations

Before interpreting the findings, this study has its limitation that must be considered. Since the study was done based on a cross-sectional study design, it did not establish the possible cause and effect relationship between independent and dependent variables. There might be potential recall bias among respondents answering questions relating to events that had previously occurred. As the survey was conducted during a dry season, it was difficult to ascertain the seasonal variations.

## Conclusion

The finding of this study demonstrated that the prevalence of wasting and stunting among HIV-positive children was relatively high. Living in a rural area, unable to read and write of caretakers, low CD4 count, recurrent oral lesion, non-biological caretakers, and unprotected source of water were found to be significantly associated with wasting. On the other hand, history of hospital admission, recurrent oral lesion, advanced WHO clinical stage, and low CD4 count were factors significantly associated with the occurrence of stunting. Therefore, timely identification and monitoring of nutritional problems should be necessary to enhance the effectiveness of ART treatment and to prevent further related complications.

## Data Availability

The data sets used and/or analyzed during the current study are available from the Corresponding author upon reasonable request.

## Abbreviations

AIDS: Acquired Immune Deficiency Syndrome
AOR: Adjusted odds ratio
ART: Antiretroviral Therapy
CI: Confidence Interval
COR: Crud odd ratio
HAZ: Height-for-age Z-score
HIV: Human immunodeficiency Virus
MUAC: Mid Upper Arm Circumference
IRB: Institutional Review Board
OI: Opportunistic Infection
SPSS: Statistical Package for Social science
WAZ: Weight-for-Age Z-score
WHO: World Health Organization
WHZ: Weight –for- HeightZ-score
